# NOX2 Deficiency Permits Sustained Survival of *S. aureus* in Macrophages and Contributes to Severity of Infection

**DOI:** 10.3389/fimmu.2021.633629

**Published:** 2021-03-22

**Authors:** Bettina Tosetti, Beate Ward, Daniela Grumme, Marc Herb, Michael Schramm, Olaf Utermöhlen, Lukas C. Heukamp, Martin Krönke, Oleg Krut

**Affiliations:** ^1^Institute for Medical Microbiology, Immunology and Hygiene, University of Cologne, Cologne, Germany; ^2^Cologne Cluster of Excellence in Cellular Stress Responses in Aging-Associated Diseases, University of Cologne, Cologne, Germany; ^3^Center for Molecular Medicine Cologne, University of Cologne, Cologne, Germany; ^4^Institute for Hematopathology Hamburg, Hamburg, Germany; ^5^German Center for Infection Research, Bonn-Cologne, Germany; ^6^Paul-Ehrlich-Institut, Langen, Germany

**Keywords:** chronic granulomatous disease, *Staphylococcus aureus*, reactive oxygen species, antibiotic treatment, sepsis model, macrophages

## Abstract

Although the crucial role of professional phagocytes for the clearance of *S. aureus* infections is well-established, several studies indicate an adverse role of leukocytes in the dissemination of *S. aureus* during infection. Since only little is known about macrophages in this context, we analyzed the role of macrophages, and in particular reactive oxygen species deficiency, for the seeding of *S. aureus* metastases. Infection of bone marrow-derived macrophages (BMDM) with *S. aureus* revealed that NADPH oxidase 2 (NOX2-) deficient, but not NOX1- or NOX4-deficient, BMDM failed to clear intracellular *S. aureus*. Despite of larger intracellular bacterial burden, NOX2-deficient BMDM showed significantly improved survival. Intravenous injection of mice with *in vitro*-infected BMDMs carrying intracellular viable *S. aureus* led to higher bacterial loads in kidney and liver of mice compared to injection with plain *S. aureus*. An even higher frequency of liver abscesses was observed in mice infected with *S. aureus*-loaded *nox2*^−/−^ BMDM. Thus, the improved intracellular survival of *S. aureus* and improved viability of NOX2-deficient BMDM is associated with an aggravated metastatic dissemination of *S. aureus* infection. A combination of vancomycin and the intracellularly active antibiotic rifampicin led to complete elimination of *S. aureus* from liver within 48 h, which was not achieved with vancomycin treatment alone, underscoring the impact of intracellular *S. aureus* on the course of disease. The results of our study indicate that intracellular *S. aureus* carried by macrophages are sufficient to establish a systemic infection. This suggests the inclusion of intracellularly active antibiotics in the therapeutic regimen of invasive *S. aureus* infections, especially in patients with NADPH oxidase deficiencies such as chronic granulomatous disease.

## Introduction

*Staphylococcus aureus* bacteremia remains worldwide the leading cause of both community acquired and nosocomial life-threatening systemic infections. *S. aureus* strains colonizing the anterior nares were found to be a frequent cause of bacteremia (1–3). Hence, breaching of the skin barrier represents one possible first step in the onset of invasive and systemic *S. aureus* infections. Despite the availability of effective antibiotics and the achievements of intensive medical care, invasive and systemic *S. aureus* infections remain a life-threatening medical challenge. However, the pathophysiologic mechanisms enabling the dissemination of *S. aureus* to inner organs remain elusive. Professional phagocytes, especially neutrophils, are crucial for the clearance of the bacteria, as several naturally occurring genetic disorders impairing neutrophil function are associated with an increased incidence and severity of *S. aureus* infections (4). Similarly, macrophages play a crucial role in the control of *S. aureus* infection (5, 6).

*S. aureus* infections belong to the signature diseases of the inherited chronic granulomatous disease (CGD), which can be caused by several mutations in genes encoding the phagocyte NADPH oxidase NOX2 or its subunits p22^phox^, p40^phox^, p47^phox^ and p67^phox^ (7). The absence or malfunction of NOX2 in neutrophils of CGD patients results in a defective oxidative burst and impaired killing of phagocytosed microbes (8, 9). In addition, mutations of CYBC1/EROS, a protein that is essential for Gp91-p22Phox heterodimer expression, have been implicated in decreased NOX2 function and CGD (10). CGD is characterized by recurrent infections with a narrow spectrum of fungi and catalase-positive bacteria including *S. aureus* (11). The importance of professional phagocytes for the immune response against *S. aureus* is also underlined by the broad range of virulence factors enabling *S. aureus* to escape efficient recognition by the host immune system or destruction by the oxidative burst of professional phagocytes (12). In addition, *S. aureus* can survive intracellularly within neutrophils *in vitro* and *in vivo* (13–15). Indeed, bloodstream neutrophils may act as Trojan horse for *S. aureus* enabling dissemination of surviving bacteria (16).

Unlike neutrophils, the role of monocytes and macrophages in systemic infections with *S. aureus* is less clear to date. Compared to neutrophils, these types of professional phagocytes lack myeloperoxidase, produce a diminished oxidative burst and exhibit an overall lesser bactericidal activity (17–23). Given their longevity and immediate presence at the site of infection, we hypothesized that macrophages might also be important for the dissemination of *S. aureus in vivo*.

In general, tissue-resident macrophages belong to the first line of defense against invading microbes (24–26). After detection, macrophages engulf the microbes by phagocytosis and inactivate, kill and degrade them in phagolysosomes (27, 28). To this end, macrophages employ an array of directly antimicrobial mechanisms, for example, the generation of reactive oxygen species (ROS) (29–31) and reactive nitrogen species (RNS) (32, 33) and the delivery of microbicidal lysosomal acid hydrolases into maturing phagosomes (34, 35).

The interaction of *S. aureus* with macrophages is multifaceted (6, 36, 37). On the one hand, macrophages use a wide array of antimicrobial mechanisms to kill *S. aureus* (6) and, in the absence of macrophages, bacterial burden and mortality following *S. aureus* infection are markedly increased (5, 38–40). On the other hand, *S. aureus* has evolved multiple strategies to survive within, manipulate and escape from macrophages (6). While the majority of phagocytosed *S. aureus* are killed by macrophages such as Kupffer cells that filter the bloodstream, a small proportion of bacteria survives (12, 41). Surviving *S. aureus* can escape from macrophages and result in formation of micro-abscesses in the liver and, potentially, lead to dissemination throughout the host. Whether this dissemination is caused by *S. aureus* that killed and escaped from macrophages or by migrating macrophages carrying intracellular *S. aureus*, is not known.

Here, we analyzed the general capacity of *S. aureus*-infected murine BMDM to enhance the systemic dissemination of *S. aureus* metastases. Specifically, we assessed the individual role of NOX1,−2 and-4 for ROS production and the survival of both, intracellular *S. aureus* and infected host cells. The intracellular survival of *S. aureus* in neutrophils and macrophages continuously embraces the hypothesis that intracellularly active antibiotics improve invasive *S. aureus* infections (42). Although rifampicin has been shown to be especially active against intracellular *S. aureus* (43) a recent clinical study testing adjunctive rifampicin for *S. aureus* bacteremia revealed no clear benefit over standard antibiotic therapy (44). However, patients suffering CGD were not included in this study. Because NOX2-deficiency aggravates the infectious challenge caused by increased loads of intracellular *S. aureus* in BMDM, we tested adjunctive rifampicin treatment to reveal a potential benefit of targeting intracellular *S. aureus* in NOX2-related disorders like CGD.

## Materials and Methods

### Mice

6–7 weeks old female C57BL/6J mice for *in vivo* experiments were obtained from Charles River Laboratories (Sulzfeld, Germany). Gp91 phox (*nox2*^−/−^) (45) and NOX4 knockout (*nox4*^−/−^) mice (46) were kindly provided by Ralf Brandes (Goethe University Frankfurt). NOX1 knockout mice (47) were kindly provided by Karl-Heinz Krause (University of Geneva) and nmf333 mice harboring the Y121H (p22^Y121H^) point mutation in the gene encoding p22phox (48), were obtained from J. Woo (Stanford University Medical Center, Stanford).

All mice were backcrossed at least 10 times to the C57BL/6J background. Mice were kept under specific pathogen-free conditions at the animal facilities of the Medical Center of the University of Cologne. All animal experiments have been carried out with local ethical committee approval (AZ 84-02.04.2014.A013) and adhering to the guidelines of German jurisdiction and the guidelines for the welfare and use of animals in research. All efforts were made to minimize suffering of the animals.

### Bacterial Strains

*Staphylococcus aureus* strain MW2, a community acquired MRSA strain also known as USA 400, was obtained from the Network on Antimicrobial Resistance in *Staphylococcus aureus* (www.narsa.net). *S. aureus* MW2 constitutively expressing GFP under control of the spa-derived constitutive promoter was constructed by transformation of MW2 with plasmid pCN-F7-GFP (49). Wildtype MW2 was used for ROS measurements, all other experiments were conducted with MW2-GFP. MW2-GFP was selected on 5 μg/ml erythromycin on agar plates and during overnight (ON) cultures. For experiments *S. aureus* were inoculated 1:100 from ON culture into fresh Luria- Bertani (LB) broth and grown at 37°C to an OD_600_ of 0.3. Bacteria were harvested, washed, and the concentration was adjusted to 1 × 10^9^ CFU/ml in PBS.

### Isolation and Culture of Bone Marrow-Derived Macrophages

For *in vitro* differentiation of bone marrow cells into bone marrow-derived macrophages (BMDM), bone marrow was prepared from the tibias and femurs of mice from the C57/BL6 J Background since this is the background of used KO mouse lines. The erythrocytes were lysed with Tris-buffered ammonium chloride (8.3% NH_4_Cl, 0.1 M Tris). Bone marrow cells were cultured in VLE RPMI 1640 medium (Biochrom), supplemented with 10% FCS, penicillin (100 U/ml) and streptomycin (100 μg/ml), 2 mM HEPES, 200 nM sodium pyruvate and 10 ng/ml recombinant M-CSF (Peprotech) to differentiate bone marrow cells into BMDM. BMDM were used for experiments at day 8 of *in vitro* differentiation unless specified otherwise. More than 90% of these cells were F4/80^+^/Cd11^+^ BMDM as determined by flow cytometry. Antibiotics were removed 16 h prior to infection of BMDM (50).

### Measurement of ROS Production by BMDM

For ROS measurements BMDM were seeded in a density of 1 × 10^5^ cells /well in sterile white 96 well LumiNunc plates (Thermo Scientific) in antibiotic free medium 16 h before measurement. After washing cells twice with cold HBSS containing magnesium sulfate (200 mg/L) and calciumchloride (185.4 mg/L) (Sigma), either viable non-opsonized or opsonized *S. aureus* MW2 [5% normal mouse serum (NMS)] were added to respective wells using MOI50 or MOI10 as indicated. Cells treated with 5% normal mouse serum (NMS; Innovative research) in HBSS served as non-infected control. Upon addition of bacteria or mouse serum, infection was synchronized by centrifugation at 840 g at 4°C in a swing bucket rotor for 5 min. Subsequently supernatant was aspirated and fresh HBSS was added to the cells. 2x Luminol/HRP mix in HBSS was added to each well. Finally, 10 μM PMA (Sigma) as positive control or 100 μg/ml Pam2CSK4 (Invivogen) as control for TLR2 dependent ROS production in HBSS was added immediately before measurement. ROS production was measured in a plate reader preheated to 37°C in 60 s intervals over 60 min (Tristar, Berthold Instruments). For evaluation first cell-free values were subtracted from each sample, subsequently values of non-infected or untreated samples were subtracted. Plotted was the corrected RLU per minute in mean and SD of triplicates.

### Assessment of Opsonophagocytic Clearing of *S. aureus* Using Flow Cytometry

To assess uptake and clearing of bacteria in BMDM, cells were harvested and adjusted to 5 × 10^6^ cells/ml. Assays were performed in triplicates using a total of 2.5 × 10^6^ cells/ml. BMDM were infected with *S. aureus* MW2 GFP (MOI15) opsonized with 5% NMS in suspension. After addition of bacteria, samples were mixed and infection was synchronized by centrifugation at 840 g at 4°C for 2 min, subsequently incubated for 5 min at 37°C rotating end over end, and thereafter the pellets were resuspended. This scheme was performed thrice. Remaining extracellular *S. aureus* were removed with Lysostaphin (Sigma; 2.5 mg/ml) in a final concentration of 0.2 mg/ml on ice for 20 min, followed by washing with PBS twice. The pellets were resuspended in 1 ml of culture medium without antibiotics and 50 μl of samples were analyzed directly (t0) and at additional time points (30 min, 1 h, 6 h and 24 h p. i.) for FACS analysis. Bacterial clearing was determined by calculating the amount of GFP positive macrophages (percentage within M1; see supp. Supplementary Figure 1) of respective time points in percent of t0. Samples were incubated rotating end over end at 37°C. After the 6 h time point Gentamycin (Sigma; 50 μg/ml) was added to the samples, to enable assessment of viability 24 h post infection. BMDM viability was assessed by trypan blue exclusion using the Countess (Life Technologies) and was calculated as percent of viable non-infected BMDM.

### Infection of Mice

BMDM on day 8 of differentiation were infected with *S. aureus*-GFP using MOI30, which results in close to 100% loading of BMDM (Supplementary Figure 1B). After Lysostaphin treatment, BMDM were washed and 2.5 × 10^6^ cells in 300 μl were administered intravenously into the tail vein of mice. 5 h post infection the amount of *S. aureus* present in infected cells was determined after lysis of BMDM in H_2_O at pH11 and subsequent plating in 10x serial dilutions on agar plates. Control mice were injected with 4 × 10^6^ plain *S. aureus* corresponding to the CFUs retrieved from *S. aureus* infected BMDM.

For determination of the bacterial load, mice were euthanized and sacrificed by cervical dislocation 24 h, 72 h and on day 7 post injection. Organs were homogenized in 0.1% Triton X-100 in gentleMACS M tubes using the predefined program for protein isolation using the gentleMACS (Miltenyi Biotec) and plated in 10-fold serial dilutions on Mueller-Hinton agar plates using the Eddy Jet spiral plater (IUL Instruments) in mode log50. After overnight incubation of the plates at 37°C, the CFUs were counted using the Countermat flash plate reader (IUL Instruments).

### Statistical Analysis

*In vitro* results and log transformed CFU values of *in vivo* experiments were analyzed by unpaired two-sided students *t*-test or 1-way ANOVA with Bonferroni post-test between selected pairs (viability data in **Figures 2E–G**). Contingency table analysis was performed using fisher' exact test. Statistical analysis and plotting of data was performed using GraphPad Prism 5.01.

## Results

### *S. aureus*-Induced ROS Production Depends on Functional NOX2 and Is Independent of TLR2

**Figure 1:**
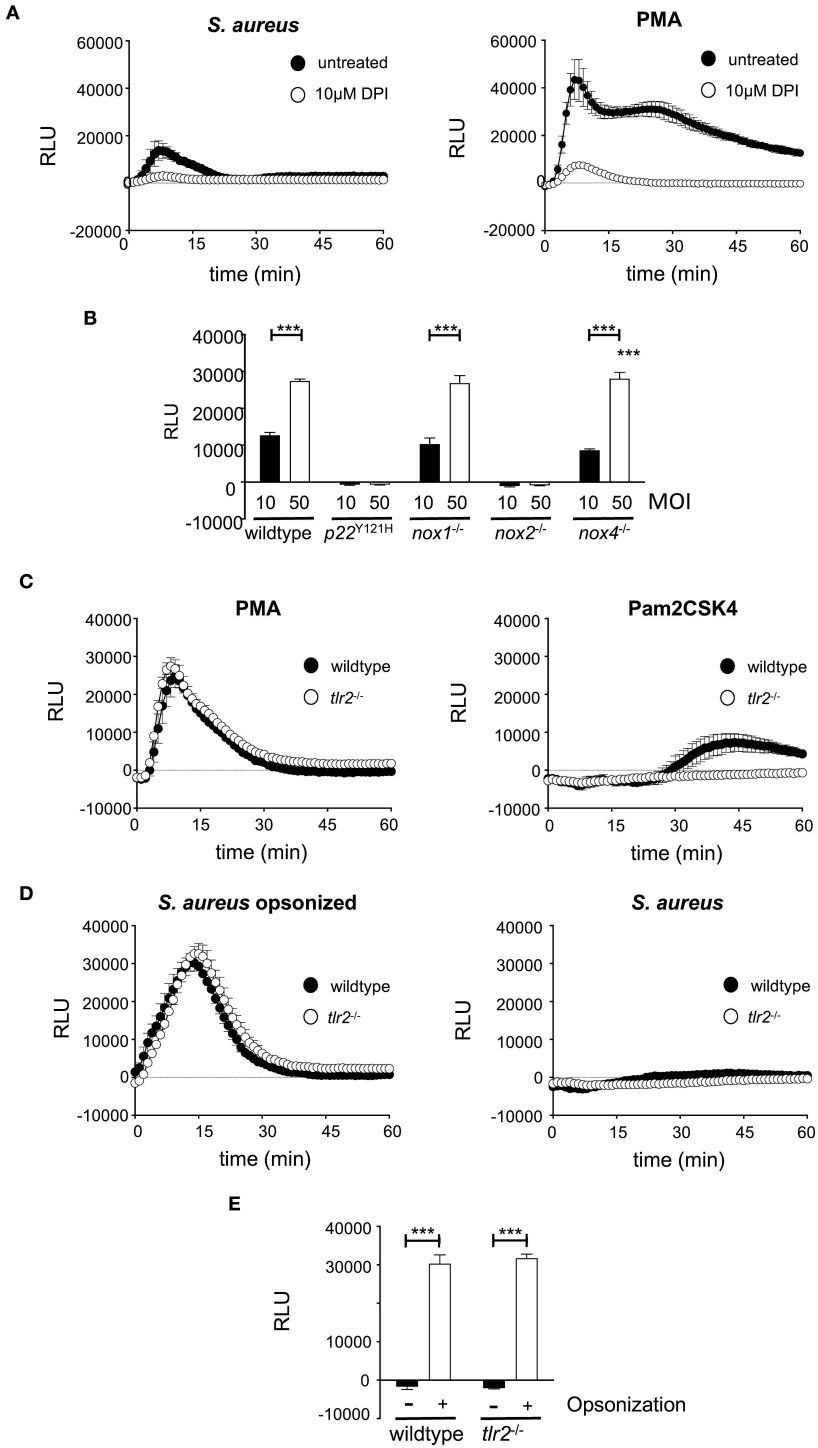
Opsonophagocytosis of *S. aureus* leads to NOX2 dependent ROS production in macrophages. For ROS measurement, 1 × 10^5^ wildtype or knockout BMDM were seeded in a white 96-well plate without antibiotics 16 h prior to measurement. ROS production was measured using Luminol in a Luminometer preheated to 37°C. **(A)** ROS production in wildtype BMDM incubated with opsonized *S. aureus* (MOI50; left) or 10μM PMA (right) in the absence (closed circles) or presence of 10 μM DPI. **(B)** Peak value of ROS production at 13 min after infection with opsonized *S. aureus* MOI10 (closed bars) or MOI50 (open bars) in wildtype and NADPH-oxidase deficient BMDMs. Shown are mean and SD of triplicates corrected for non-infected control of a representative experiment performed at least three times. Statistical significance was analyzed by unpaired two-sided Student's *t*-test using GraphPad Prism 5.01 between indicated conditions (^***^=*p* < 0.001). **(C)** ROS production in wildtype (closed circles) or *tlr2*^−/−^ BMDM (open circles) stimulated with 10 μM PMA (left), or 100 ng/ml Pam_2_CSK_4_ (right). **(D)** ROS production in wildtype (closed circles) or *tlr2*^−/−^ BMDM (open circles) upon infection with *S. aureus* MW2 opsonized with 5% mouse serum (MOI50; left) or non opsonized *S. aureus* (MOI50; right). **(E)** Peak value of ROS production at 13 min after infection with non-opsonized (closed bars) or opsonized *S. aureus* (open bars) in wildtype and *tlr2*^−/−^ BMDM. Shown are means and SD of triplicates of a representative experiment performed at least three times. Statistical significance was analyzed by unpaired two-sided Student's *t*-test using GraphPad Prism 5.01 between indicated conditions (^***^=*p* < 0.001). NOX, NADPH Oxidas; ROS, reactive oxygen species; PMA, phorbol 12-myristate 13-acetate; DPI, diphenyleniodonium; MOI, multiplicity of infection; BMDMs, bone marrow derived macrophages; SD, standard deviation; TLR2, Toll-like receptor 2; NMS, normal mouse serum; p.i., post infection; CFU, colony forming unit; i.v., intravenous; s.c., subcutaneous.

Macrophages do not always succeed in *S. aureus* killing, thereby providing a niche for persistence, which fosters continued infection (36). It has been speculated for a long time that professional phagocytes might serve as Trojan horses for *S. aureus* and lead to the frequently observed dissemination from the primary focus of infection (15). Whereas the role of neutrophils has been evaluated in greater detail, macrophages, although being a source of bacterial persistence, have been far less studied. A prerequisite for bacterial persistence is a perfect symbiosis of intracellular *S. aureus* and host cells, which can only be achieved when the bacteriocidal activities of macrophages are kept at bay. One of the most important bacteriocidal mechanisms of macrophages, the oxidative burst is generated by the NOX family of NADPH oxidases. Next to NOX2, at least two additional NOX isoforms, namely NOX1 and NOX4, are expressed by macrophages and might be involved in ROS generation. Indeed, wildtype BMDM strongly responded with ROS production to opsonized *S. aureus* (Figure 1A, left). The kinetics of ROS production was comparable to that of BMDM stimulated pharmacologically with 10 μM PMA used as positive control (Figure 1A, right). Treatment with the NOX inhibitor DPI led to a significant inhibition of ROS production (Figure 1A). We next scrutinized the relative contribution of NOX-isoenzymes 1, 2 and 4 to the oxidative burst of macrophages in response to *S. aureus*. To this end, BMDM with genetically defined deficiencies for individual NOX isoforms were employed, including *nox1*^−/−^*, nox2*^−/−^ *and nox4*^−/−^ as well as *p22*^*Y*121*H*^, a non-functional mutant of the NADPH oxidase subunit p22^phox^ that is common to isoforms NOX1,−2,−3, and−4. The oxidative burst induced by *S. aureus* in BMDM from *nox1*^−/−^ and *nox4*^−/−^ mice was MOI-dependent and comparable to that of wildtype BMDM (Figure 1B). By contrast, macrophages isolated from *nox2*^−/−^ or *p22*^*Y*121*H*^ mice showed completely abolished production of *S. aureus*-induced ROS. These findings demonstrate that mainly the NADPH oxidase NOX2 is responsible for ROS production by BMDM upon *S. aureus* infection and raised the question about the control of NOX2 activation.

NOX2 is activated by opsonophagocytic receptors like FCγR and macrophage-1 antigen (Mac-1) (36, 51). Indeed, opsonized *S. aureus* led to strong ROS production (Figures 1C,D). Infection with non-opsonized *S. aureus* did not stimulate any measurable production of ROS at this time after infection (Figure 1D). TLR2-dependent activation of JNK results in inhibition of ROS production upon *S. aureus* infection and increased bacterial survival (52). Pam_2_CSK_4_, a synthetic TLR2 ligand, did not induce TLR2-dependent ROS production within the first 30 min (Figure 1C). Furthermore, opsonized *S. aureus* induced comparable production of ROS in either TLR2-deficient or -proficient BMDM (Figures 1C–E). Thus, the induction of NOX2-mediated ROS production by phagocytic receptors seems to overrule any possibly antagonistic action of TLR2.

### Elimination of Phagocytosed *S. aureus* and Survival of Infected BMDM Depend on NOX2 Activity

**Figure 2:**
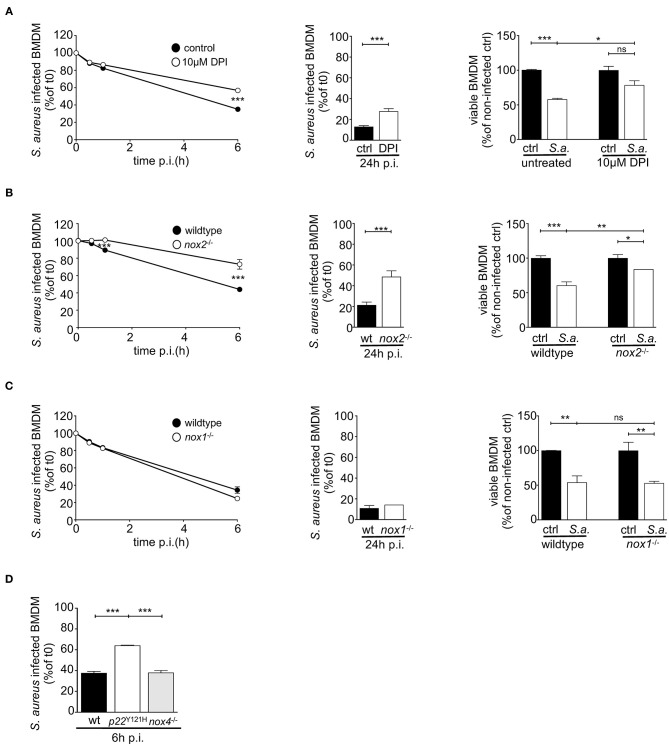
*S. aureus* clearing and survival of infected BMDM depend on functional NOX2. BMDM were infected with GFP-expressing *S. aureus* MW2 (MOI15) opsonized with 5% NMS in suspension and assessed 24 h p.i. by flow cytometry for clearing of *S. aureus* and for BMDM viability (right panel). **(A)** BMDM pre-treated with 10 μM DPI (open circles) or left untreated (closed circles), **(B)** wildtype (closed circles) and *nox2*^−/−^ BMDM (open circles), **(C)** wildtype (closed circles) and *nox1*^−/−^ BMDM (open circles). Statistical significance was analyzed by unpaired two-sided Student's *t*-test using GraphPad Prism 5.01 between indicated conditions (^**^ = *p* < 0.01; ^***^= *p* < 0.001). The viabilities of BMDMs at 24 h p.i. were assessed by trypan blue exclusion and depicted as bar charts (**A–C**, right). Represented is the percentage of viable cells referring to corresponding non-infected cells for each genotype in mean and SD of triplicates. Statistical significance was analyzed by 1-way ANOVA with Bonferroni post-test between selected pairs as implemented in GraphPad Prism 5.01 between indicated conditions (^*^= *p* < 0.05; ^**^ = *p* < 0.01; ^***^= *p* < 0.001). **(D)** Clearing kinetics of wildtype, p22phox^Y121H^ and *nox4*^−/−^ at 6h post infection. Shown are means and SD of triplicates as percent of corresponding values at t0. Shown are representative experiments performed three times. NOX, NADPH Oxidas; ROS, reactive oxygen species; PMA, phorbol 12-myristate 13-acetate; DPI, diphenyleniodonium; MOI, multiplicity of infection; BMDMs, bone marrow derived macrophages; SD, standard deviation; TLR2, Toll-like receptor 2; NMS, normal mouse serum; p.i., post infection; CFU, colony forming unit; i.v., intravenous; s.c., subcutaneous.

To investigate the impact of the reduced oxidative burst in NOX2-deficient BMDM on the survival of both, intracellular *S. aureus* and host cell, we determined the kinetics of relative numbers of *S. aureus*-positive BMDM and the viability of host cells. ROS inhibition in BMDM by treatment with DPI significantly impaired the clearance of intracellular *S. aureus* over the first 24 h (Figure 2A). At 24 h post infection about 25% of DPI-treated BMDM were positive for GFP-expressing *S. aureus*, whereas only 10% of untreated BMDM carried *S. aureus*. As expected, NOX2-deficient BMDM, like DPI-treated BMDM, showed a markedly impaired clearance of intracellular *S. aureus* (Figure 2B). In contrast, *nox1*^−/−^ and *nox4*^−/−^ BMDM cleared *S. aureus* like wildtype BMDM (Figures 2C,D), which corresponds to their normal ability to elicit an oxidative burst. Thus, ROS production by NOX2 in BMDM is a prerequisite for the ability to clear phagocytosed *S. aureus*.

The question arose whether the reduced relative numbers of *S. aureus*-positive BMDM result from successful ROS-dependent killing of intracellular *S. aureus* or rather are secondary to killing of the BMDM by *S. aureus*. Therefore, we investigated the viability of BMDM. DPI-treated as well as *nox2*^−/−^ BMDM had a substantial advantage in survival upon infection, despite their higher bacterial burden (Figures 2A–C; right). In fact, the viability of non-infected wildtype BMDM was less than 60% indicating that *S. aureus* infection led to cell death in 40% of infected BMDM and suggesting that *S. aureus* may have escaped into the culture supernatant and subsequently killed by gentamycin present in the culture medium. This scenario is consistent with a continuous cycle of phagocytosis, intracellular *S. aureus* replication, host cell death, bacterial release and re-uptake by macrophages (which may only occur in the absence of antibiotics) as recently proposed by Jubrail and colleagues (37, 53). In contrast, *S. aureus* induced cell death in only 15% of NOX2-deficient or DPI-treated BMDM (Figures 2A,B; right). Together, these data suggest that the oxidative burst not only reduces the number of BMDM carrying phagocytosed *S. aureus* but also contributes to death of infected macrophages. Furthermore, a defective oxidative burst likely renders BMDM an intracellular niche for *S. aureus* survival and replication.

### NOX2 Deficiency Enhances Dissemination of *S. aureus* by Macrophages

**Figure 3:**
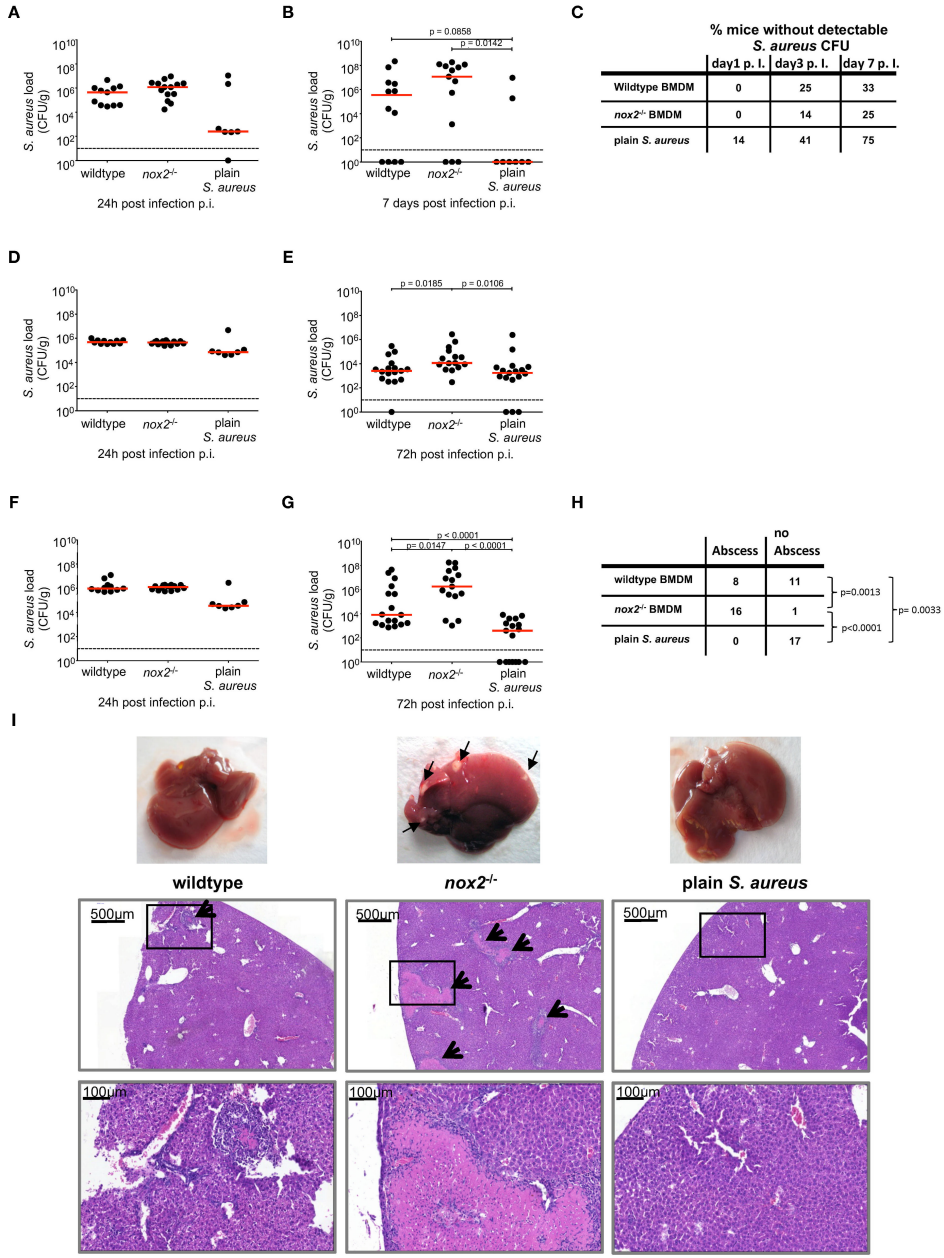
Bacterial load in kidney, spleen and liver from mice upon injection of BMDM infected with *S. aureus*. BMDM of wildtype or *nox2*^−/−^ mice were infected with *S. aureus* MW2 GFP (MOI30) on day 8 post isolation from bone marrow. Upon infection 2.5 × 10^6^ BMDM were injected in PBS intravenously into naive C57Bl6J mice. Mice injected with 4 × 10^6^ plain *S. aureus* MW2 GFP, corresponding to the CFU recovered from infected BMDM served a control. Represented are individual values for each organ in CFU/g from two independent experiments. Red line indicates median, dashed line represents limit of detection. Student's *t*-test was performed on log-transformed values. CFU numbers were analyzed. in kidneys **(A)** 24 h (wt: n=11; *nox2*^−/−^: *n*=14; plain *S. aureus*: *n*=7) and **(B)** 7 days (wildtype: *n*=12; *nox2*^−/−^: *n*=13; plain *S. aureus*: *n*=8). **(C)** Percentage of mice without detectable CFU in kidneys for indicated timepoints. CFU numbers in spleen **(D)** 24 h and **(E)** 72 h (wildtype: n=17; *nox2*^−/−^: n=15; plain *S. aureus*: *n*=17) p.i. CFU numbers in liver **(F)** 24 h and **(G)** 72 h p.i. **(H)** Contingency table for presence or absence of visible abscesses in liver 72 h post infection analyzed by Fisher‘s exact test. **(I)** Representative pictures from livers 72 h post infection with micrographs from respective Hematoxylin-Eosin staining on formalin fixed tissue. Black arrows indicate abscesses. Scale bar represents 500 μm in upper and 100 μm in lower micrographs, respectively. Rectangular zone in upper pictures indicates position of bottom pictures. NOX, NADPH Oxidas; ROS, reactive oxygen species; PMA, phorbol 12-myristate 13-acetate; DPI, diphenyleniodonium; MOI, multiplicity of infection; BMDMs, bone marrow derived macrophages; SD, standard deviation; TLR2, Toll-like receptor 2; NMS, normal mouse serum; p.i., post infection; CFU, colony forming unit; i.v., intravenous; s.c., subcutaneous.

The survival of *S. aureus* inside BMDM and in particular the prolonged survival of *nox2*^−/−^ macrophages observed in our experiments *in vitro* raised the question about possible functional consequences *in vivo*, such as enhanced pathogenicity and dissemination of *S. aureus* in mice. Therefore, we next investigated the course of infection in mice challenged either with *S. aureus* internalized by macrophages or with the corresponding dose of plain *S. aureus* injected as a bacterial suspension. Wildtype or *nox2*^−/−^ BMDM were infected *in vitro* with *S. aureus* and subsequently administered i.v. into wildtype C57BL/6J mice. The dose of plain *S. aureus* administered as a bacterial suspension corresponded to the number of live *S. aureus* recovered from infected *nox2*^−/−^ BMDM (4 × 10^6^ CFU). Mice challenged with BMDM loaded with intracellular *S. aureus* showed larger CFU counts in kidneys and liver, and less prominently in spleen, on day 7 after infection than mice challenged with plain *S. aureus* (Figures 3A–G). Notably, when *nox2*^−/−^ BMDM were used as vehicles, they produced by trend the highest bacterial load in kidneys and liver. Intravenous injection of plain *S. aureus* also induced bacterial dissemination in mice. Animals infected by plain bacteria more efficiently cleared *S. aureus* from kidney and liver than animals injected with BMDM-associated *S. aureus* (Figures 3B,C,G–I).

Macroscopical examination of explanted livers revealed that all livers from mice injected with infected *nox2*^−/−^ BMDM contained one or more subcapsular abscesses of at least 1 mm in diameter (Figure 3I). Therefore, we assessed the frequency of subcapsular liver abscesses at 72 h post infection in the three experimental groups. The incidence of liver abscesses was significantly increased in the group that received *nox2*^−/−^ BMDM carrying *S. aureus* as compared to the group that received wildtype BMDM carrying *S. aureus* (Figure 3H). No subcapsular abscesses were detected in livers of mice that received plain *S. aureus*. Histological analysis of HE-stained sections revealed relatively small abscesses (about 1.5 × 10^4^ μm^2^) for mice that received wildtype BMDM carrying *S. aureus* (Figure 3I). By contrast, liver sections of mice that received *nox2*^−/−^ BMDM carrying *S. aureus* revealed large abscesses with extensive necrosis and excessive inflammatory infiltrates. In one field of view, in total eight abscesses could be detected, which had an average area of about 3 × 10^5^ μm^2^. As expected, in the livers from mice receiving plain *S. aureus*, no histopathological signs of infection were detected, consistent with the relatively low CFU count at 72 h post infection. These data suggest that intracellular *S. aureus* internalized by macrophages induce a disseminated and long-lasting infection in mice, especially when compared to plain bacteria. Moreover, the aggravation of the infection caused by *nox2*^−/−^ BMDM-associated *S. aureus* underscores the pathogenic potential of a weakened but surviving host cell to provide a niche for *S. aureus* survival and replication.

### Antibiotic Targeting of Intracellular *S. aureus* Ameliorates the Course of Infection

**Figure 4:**
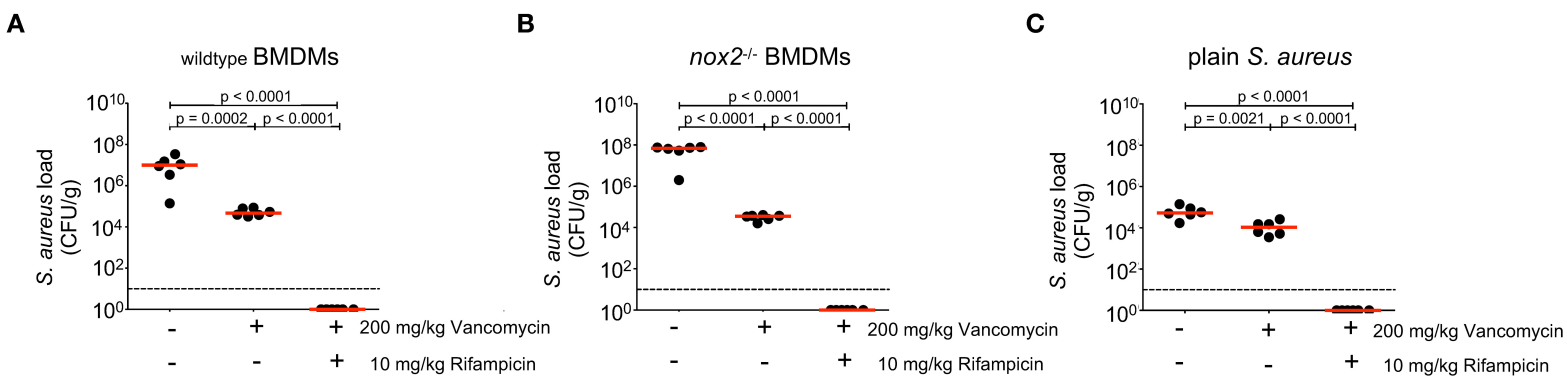
Effects of antibiotic treatment on bacterial load in organs 24 h upon injection of BMDM infected with *S. aureus*. BMDM of **(A)** wildtype or **(B)** *nox2*^−/−^ mice were infected with *S. aureus* MW2 GFP (MOI30). 2.5 × 10^6^ infected BMDMs were injected in PBS i.v. into naive C57Bl6J mice (*n*=6 per genotype and treatment). **(C)** Mice injected with 4 × 10^6^ plain *S. aureus* MW2 GFP, corresponding to the CFU recovered from infected BMDM (*n*=6 per treatment). 200 mg/kg vancomycin was injected s.c. either alone or in combination with 10 mg/kg Rifampicin at 1 h, 6 h, 24 h, and 30 h after i.v. infection. At 48 h p.i. livers of infected mice were homogenized and plated on Mueller-Hinton plates. Shown are individual values for each liver in CFU/g. Red line indicates median, dashed line represents limit of detection. Student's *t*-test was performed on log-transformed values. NOX, NADPH Oxidas; ROS, reactive oxygen species; PMA, phorbol 12-myristate 13-acetate; DPI, diphenyleniodonium; MOI, multiplicity of infection; BMDMs, bone marrow derived macrophages; SD, standard deviation; TLR2, Toll-like receptor 2; NMS, normal mouse serum; p.i., post infection; CFU, colony forming unit; i.v., intravenous; s.c., subcutaneous.

*S. aureus* infections are treated with antibiotics like ß-lactams or vancomycin that are extracellularly active, sometimes in combination with intracellularly active antibiotics like clindamycin or rifampin. Our results suggest that BMDM carrying viable *S. aureus* either die and release *S. aureus* into the extracellular milieu, where they are exposed to antibiotics. BMDM carrying viable *S. aureus* are yet also able to survive and initiate and maintain an infection with enhanced severity *in vivo*. We therefore hypothesized that a combined treatment with an extracellularly active antibiotic together with an intracellularly active antibiotic should be superior to treatment with an extracellularly active antibiotic alone. To test this hypothesis, mice were challenged with *S. aureus*-containing wildtype BMDM (Figure 4A), *S. aureus-*containing *nox2*^−/−^ BMDM (Figure 4B) or with plain *S. aureus* (Figure 4C). After 1 h of infection, mice subcutaneously received the extracellularly active antibiotic vancomycin alone, or in combination with the intracellularly active rifampicin. Rifampicin was chosen because of its excellent ability to eradicate intracellular *S. aureus* in host cells (43). Injection of antibiotics was repeated at 6, 24 and 30 h post infection to account for the limited half-life of vancomycin. At 48 h p.i., livers were explanted and analyzed for bacterial loads. First, we observed that wildtype and *nox2*^−/−^ BMDM carrying *S. aureus* produced 2–3 logs greater numbers of *S. aureus* (CFU/g) than plain *S. aureus* (Figure 4, compare A, B, and C), which is probably the result of almost instant exposure of extracellular *S. aureus* to the bacteriocidal antibiotic vancomycin and to the innate host defense. In the two BMDM groups, vancomycin alone reduced the bacterial load in livers in a statistically significant manner (Figures 4A,B) suggesting that *S. aureus* must escape BMDM over time and are subsequently eliminated by exposure to vancomycin. When mice were infected with plain *S. aureus*, vancomycin reduced the bacterial burden to a lesser, although still significant, extent leaving a large residual population of viable *S. aureus* particularly in the liver (Figure 4C). This finding suggests that a large proportion of plain *S. aureus* are protected from vancomycin *in vivo*, probably by internalization into professional phagocytes. Indeed, the combined treatment of mice with vancomycin and rifampicin resulted in complete elimination of *S. aureus* (Figures 4A–C), which underscores a potential benefit of intracellularly active antibiotics in the treatment of *S. aureu*s infections.

## Discussion

Although macrophages play a crucial role in the control of *S. aureus* infection (5), macrophages do not always succeed in *S. aureus* killing, thereby providing a reservoir for bacterial persistence (36). It has been speculated for a long time that neutrophils might serve as Trojan horses for *S. aureus* and drive dissemination from the primary focus of infection (15). However, whereas the role of neutrophils has been evaluated in greater detail, macrophages, although being a source of bacterial persistence, have been far less studied. We here show that the NADPH oxidase NOX2 plays a crucial role in determining the survival of both intracellular *S. aureus* and infected bone marrow-derived macrophages. Furthermore, NOX2-deficient BMDM carrying viable intracellular *S. aureus* were important drivers of systemic dissemination of the pathogen and aggravation of infection. Eventually, *in vivo* experiments reveal that rifampicin in adjunction of vancomycin completely eradicates *S. aureus* from liver, kidneys and spleen suggesting a therapeutical potential, targeting *S. aureus*-carrying macrophages in systemic *S. aureus* infections, especially in NOX2 deficiency related disorders.

Based on the early observations of Rogers and colleagues and clinical data from the UK, neutrophils were proposed as Trojan horses for the metastasis of *S. aureus* infection (12, 15, 54, 55). Gresham and co-workers were able to show that *S. aureus*-infected murine neutrophils obtained from the peritoneal cavity at 24 h post infection readily establish infection when transferred into healthy recipient mice (13). However, neutrophils are short-lived cells and die rapidly when unable to control *S. aureus* infection (14, 56). Several studies analyzed intracellular survival of *S. aureus* in macrophages (36, 37, 57–60). Since macrophages, amongst others, eliminate apoptotic neutrophils and were shown to play an important role in clearance of *S. aureus* upon local infection of the lungs (61), these long-lived cells are also suited as Trojan horse.

Kubica et al. observed that some *S. aureus* survive for several days in human monocyte-derived macrophages before eventually lysing the host cell and subsequently undergo extracellular replication (60). More recently, Jubrail et al. have shown that, although intracellular antimicrobial mechanisms initially kill *S. aureus* rapidly after ingestion, they become progressively exhausted despite ongoing phagocytosis leading to host cell lysis and re-uptake by macrophages, which ultimately results in an intracellular pool of persisting *S. aureus* (37). In line with their observations, our present study provides *in vitro* evidence that *S. aureus* is able to survive within BMDM despite the presence of functional NOX2. As expected, NOX2-deficient macrophages were even more permissive, showing a markedly improved survival upon infection *in vitro*.

ROS represent only one among many other mechanisms to destroy phagocytosed pathogens, which include RNS, proteolytic enzymes, antimicrobial peptides, as well as acidification of the phagolysosome, nutrient restriction, autophagy or extracellular traps. However, mutations in lysosomal enzymes (e.g., myeloperoxidase) processing superoxide radicals into highly active antimicrobial compounds like hypochlorite are less associated with *S. aureus* infections than NADPH oxidase deficiency (62). Indeed, the outstanding role of NOX2 for the containment of *S. aureus* infections is reflected by CGD, a genetic immune deficiency affecting the function of the phagocyte NADPH oxidase NOX2, where *S. aureus* infection is one of the signature complications (8). Although ROS are major, directly acting defense factors against bacteria, other indirect ROS-dependent events severing intracellular processing of *S. aureus* have been postulated, such as the antagonism of v-ATPase-mediated proton influx inhibiting phagosomal acidification and maturation (37, 63). In addition, the link between Nox2-derived ROS production and the induction of a highly bactericidal form of phagocytosis called LC3-associated phagocytosis was recently discovered and is crucial for degradation of many pathogens (31, 40, 64–67). These reports are in line with our observation that *nox2*^−/−^ BMDM are more permissive for intracellular *S. aureus* than wildtype BMDM resulting in better survival of both *S. aureus* and host cells, which in turn leads to greater metastatic dissemination of *S. aureus*.

This study has some limitations. Although bone marrow cells were differentiated *in vitro* using M-CSF, which drives differentiation into the macrophage lineage, it is not clear whether these *in vitro*-generated BMDM reflect natural macrophages in all their functional facets such as *in vivo* trafficking, self-renewal potential in specific tissues, polarization into pro- or anti-inflammatory phenotypes and anti-bacterial defense. Furthermore, only few *S. aureus* strains were investigated in a single strain of mice (C57BL/6J), which does not allow for generalizations. However, this study is consistent with and extends previous excellent reports from many other laboratories, who analyzed the interaction of macrophages and *S. aureus* in great detail (36, 37), for review (6, 53). In this study, we recapitulated these observations with BMDMs of different genotypes and found no reason to believe that BMDM were fundamentally different to *in vivo* differentiated macrophages in this context.

Evidence from previous work and the present study is accumulating, indicating that wildtype macrophages, and especially NOX2-deficient macrophages, provide a protective niche for *S. aureus* enabling the dissemination and aggravation of infection. As to clinical practice, an efficient antibiotic treatment strategy should address intracellular *S. aureus*, which is especially important in CGD, where macrophages lack NOX2 function (8). Indeed, the ISDA Clinical Practice Guidelines recommend adjunctive rifampicin in specific instances at the B-III level, meaning there is moderate evidence to support a recommendation for or against use, where the evidence comes from opinions of respected authorities, based on clinical experience or descriptive studies, or reports of expert committees (68). A more recent clinical study testing adjunctive rifampicin for *S. aureus* bacteremia revealed no clear overall benefit over standard antibiotic therapy (44). Although subgroup analyses suggested some benefit in those groups with methicillin-sensitive *S. aureus* infection treated with flucloxacillin as the only backbone antibiotic (*p*=0.01), the authors concluded that, with 20 subgroups analyzed, one statistically significant association might have occurred by chance (44). Whereas the clinical use of rifampicin remains controversial, rifampicin is intracellularly effective even within the acidic vacuoles of neutrophils (69) as well as in non-phagocytic cells (43). We show here that a combination therapy of vancomycin and rifampicin in mice infected intravenously with either intracellular *S. aureus* inside wildtype BMDM or *nox2*^−/−^ BMDM or with plain bacteria resulted in complete clearance of S. *aureus* from the liver. In contrast, mice receiving vancomycin alone only reduced the CFU number and the remaining bacterial loads still ranged between 1 × 10^4^ and 1 × 10^5^ CFU/g. Thus, it still might be a worthwhile clinical study to test adjunctive rifampicin treatment of invasive *S. aureus* infections, especially in patients with CGD.

## Data Availability Statement

The original contributions presented in the study are included in the article/Supplementary Material, further inquiries can be directed to the corresponding author/s.

## Ethics Statement

The animal study was reviewed and approved by Ethics commitee University of Cologne (AZ 84-02.04.2014.A013).

## Author Contributions

BT, MK, and OK planned and supervised the project. OK, BT, MS, BW, and DG performed experiments. LH performed histopathology. BT, OK, MS, and OU wrote manuscript. MK, MS, MH, BT, and OK revised the manuscript. All authors contributed to the article and approved the submitted version.

## Conflict of Interest

The authors declare that the research was conducted in the absence of any commercial or financial relationships that could be construed as a potential conflict of interest.

## Supplementary Material

Supplementary Figure 1 Flow cytometry analysis of *S. aureus* clearing in BMDMs. BMDMs were infected with GFP-expressing *S. aureus* MW2 opsonized with 5% NMS and assessed for clearing by flow cytometry. **(A)** representative dot-plot to demonstrate gating strategy of BMDM population. 5000 events within gate R1 were measured and the percentage of GFP positive BMDMs within this gate was assessed by histogram plot analysis of counts versus channel FL1-H (GFP fluorescence). **(B)** representative histograms of wildtype (left) or NOX2-/- BMDMs infected with MOI 1.5, MOI15 and MOI30 to define conditions for optimal loading and clearing assays. M1, this marker represents the gate for GFP positive BMDMs. The grey filled plot represents non infected BMDMs. Fluorescence of infected BMDM samples was color-coded corresponding to specific time points p.i.: t0, red line; t6h p.i., dark blue line; and t24h p.i., light blue line. The corresponding fluorescence values are presented in plots. T0 reflects the time point after infection followed by immediate lysis of extracellular *S. aureus* by lysostaphin. **(C)** Representative histograms of BMDMs infected with an MOI15 of GFP-expressing *S. aureus* MW2. These plots correspond to results as shown in Figures 2A–D, respectively. Note different scale of Y-axis for the top panel.
